# Hepatitis B Seroprevalence in the Pediatric and Adolescent Population of Florence (Italy): An Update 27 Years after the Implementation of Universal Vaccination

**DOI:** 10.3390/vaccines8020156

**Published:** 2020-03-30

**Authors:** Beatrice Zanella, Angela Bechini, Sara Boccalini, Gino Sartor, Emilia Tiscione, Working Group DHS, Working Group AOUMeyer, Working Group AUSLTC, Paolo Bonanni

**Affiliations:** 1 Department of Health Sciences, University of Florence, 50134 Florence, Italy; beatrice.zanella@unifi.it (B.Z.); angela.bechini@unifi.it (A.B.); sara.boccalini@unifi.it (S.B.); emilia.tiscione@unifi.it (E.T.); 2 Medical Specialization School of Hygiene and Preventive Medicine, University of Florence, 50134 Florence, Italy; gino.sartor@unifi.it (G.S.); federico.manzi@unifi.it; 3 Meyer Children’s Hospital, 50139 Florence, Italy; francesco.puggelli@meyer.it; 4 AUSL Toscana Centro, 50142 Florence, Italy; lorenzo.baggiani@uslcentro.toscana.it

**Keywords:** hepatitis B, vaccination, seroprevalence, ELISA test, Italy

## Abstract

Background: Hepatitis B still represents a health concern, although safe and effective vaccines have been available since 1982. Italy introduced a program of universal vaccination against hepatitis B in 1991. The aim of this study was to assess the immunity levels towards hepatitis B in a sample of sera from the pediatric and adolescent population in the province of Florence, Central Italy, twenty-seven years after the implementation of universal vaccination. Methods: A total of 165 sera samples were collected from the resident population of Florence aged 1–18 years. The anti-HBs and anti-HBc enzyme-linked immunosorbent Assay (ELISA) tests were performed on all samples. The anamnestic and vaccination status data were also collected. Results: Seroprevalence of anti-HBs was approximately 60%, with children aged 1–5 years having the highest positivity rate (81.6%), and decreasing trends in the older age groups. The zero prevalence of anti-HBc shows that the detected protective immunity is mainly due to vaccination, and natural infection was not reported in the studied population. Conclusions: The seroprevalence of anti-HBs and the lack of anti-HBc in this study highlights that immunity levels have been derived mainly from immunization. This confirms how vaccination dramatically reduced circulation of the hepatitis B virus in Italy in the pediatric and adolescent population twenty-seven years after implementation of the mandatory universal program.

## 1. Introduction

Hepatitis B is a serious infection due to the hepatitis B virus (HBV), which infects the hepatocytes causing an acute or a chronic disease. It is still a major public health concern due to the high risk of death from chronic infection sequelae, like cirrhosis and liver cancer. The World Health Organization (WHO) has estimated 257 million people are living with the HBV infection, and in 2015, the infection caused 887,000 deaths, mainly from its complications [1]. The virus is highly contagious and spreads through contact with blood or other body fluids (sperm or vaginal fluids) of an infected subject. Furthermore, the HBV manages to survive outside the body, for example, on surfaces, even for months under proper conditions, and for this reason it also represents a significant occupational hazard for healthcare workers [1,2]. Safe and effective vaccines against hepatitis B have been available since 1982; they prevent both the infection and the development of chronic diseases and their related complications [1].

Hepatitis B surface antigen (HBsAg) prevalence in the general population varies considerably among the WHO regions: currently, the Western Pacific Region and the Africa Region are high-intermediate endemicity areas, in which, respectively, 6.2% and 6.1% of the adult population is chronically infected. In the Eastern Mediterranean Region and the South-East Asia Region, an estimated 3.3% and 2.0% of the general population is infected, while the European Region and the Region of Americas are low endemic areas, with the lowest values of prevalence (respectively, 1.6% and 0.7%) [1,3]. However, hepatitis B prevalence is not homogeneous throughout the European Region. As a matter of fact, it ranges from very low levels (0.1%), especially in some areas in Western, Northern, and Central Europe, to high prevalence (6–8%) in some countries of Eastern Europe and central Asia [4]. In recent years, the European Centre for Disease Prevention and Control reported a decline in the rate of acute cases of hepatitis B, from 1.2 cases per 100,000 inhabitants in 2007 to 0.6 cases per 100,000 inhabitants in 2016. This result, in accordance with global trends, most likely reflects the impact of national anti-hepatitis B immunization programs [5].

Vaccination against HBV is the most cost-effective strategy of prevention: the WHO recommends the administration of the first dose of an anti-hepatitis B vaccine to all newborns within 24 h from birth that should be followed by 2 or 3 doses to complete the prime immunization series. After a three-dose vaccination schedule, vaccines induce protective levels (anti-HBs ≥ 10 mIU/mL) (mIU: milliInternational Unit) in more than 95% of infants, children, and young adults, and the protection could last more than 20 years or be lifelong. For this reason, the WHO does not recommend a booster vaccination to responder subjects that have completed the prime immunization course [1].

Italy was one of the first countries that implemented hepatitis B vaccination in the national immunization program (NIP). In 1991, a mandatory vaccination policy against hepatitis B was introduced, with a double-cohort strategy of immunization addressed to all newborns in the first year of life, and to 12-year-old adolescents [6]. In 2003, hepatitis B vaccination remained mandatory only for the newborn cohorts, due to the merging of the infant and adolescent cohorts 12 years after the implementation of the mandatory law. Recently, in 2017, the anti-HBV vaccination was included as a mandatory requirement to attend school for all subjects aged 0–16 years, including unaccompanied foreign minors [7]. In Italy, since 2013, HBV vaccination coverage (VC) with three doses at 24 months of life had started to gradually decline, reaching the historical minimum value in 2016 (92.9%). In 2017, the VC increased to 94%, although it remained below the recommended threshold (95%) [8,9,10,11,12].

In the last three decades, the Italian epidemiological landscape regarding hepatitis B has changed profoundly, with a decreasing trend of incidence and prevalence of the hepatitis B infection. Many factors have contributed to this change, such as better socioeconomic conditions and general measures, like implementation of health promotion campaigns and new blood safety strategies; a better compliance to the standard precautions and to the use of disposable health devices; and, above all, the implementation of effective vaccination programs against the HBV. The implementation of anti-HBV immunization in all infants and adolescents has played a key role in the prevention of this infection: a time series of the incidence of hepatitis B shows that the most marked decrease corresponds to the years after its introduction. In fact, the incidence of acute disease has clearly declined from 12 cases per 100,000 inhabitants in 1985 to 0.4 cases per 100,000 inhabitants in 2018. In particular, the reduction mostly involved specific age groups, like infants, adolescents, and young adults under 24 years of age [13]. Furthermore, the Italian population under 40 years, which has been actively receiving vaccination for the last 28 years, can be considered immunized and protected from both acute and chronic infection [14]. In 2018, the SEIEVA (Epidemiological Integrated System of Acute Viral Hepatitis) surveillance system showed 212 new cases of acute hepatitis B, corresponding to an incidence of 0.4 cases per 100,000 inhabitants. As in the previous years, the most affected age group is that of adults aged 35–54 years; as a matter of fact, this age group accounted for the highest incidence (0.8 cases per 100,000 inhabitants) in 2018. About 76% of acute cases are reported in the male population. Furthermore, no new cases of acute hepatitis B were detected in the younger population (0–14 years) in 2018. The most frequently reported risk factor was exposure to beauty treatments like manicures, piercings, and tattoos (35% of cases), while sexual exposure accounted for 28% of cases [15].

In Tuscany, during the period 2013–2017, the highest incidence was registered in the adult population aged 25–44 and 45–64 years (2.3 cases per 100,000 inhabitants for both age groups), with the male population mainly involved. In 2017, no cases of acute hepatitis B occurred in subjects under 25 years [16]. In 2018, the Regional Agency for Health reported 30 new cases of acute hepatitis B, corresponding to an incidence rate of 0.8 cases per 100.000 inhabitants; no cases of hepatitis B were reported among the population aged 0-4 years, while four cases occurred among the population aged 5–24 years [17]. Of those 4 cases, two occurred in unvaccinated subjects of 23 years and 21 years (the latter of Asian origin), while two other cases were reported in female subjects classified as “vaccinated” of, respectively, 14 and 19 years, but for whom no information was available on the number of doses they actually received [18].

In 2000 and 2009, two seroepidemiological surveys were carried out in the province of Florence in order to assess the impact of hepatitis B vaccination 10 years and 20 years after its implementation. The seroprevalence of anti-HBs among the population aged 1–20 years was approximately 82.4% in 2000 [19] and 58.6% in 2009 [20].

The aim of our study was to assess the immunity/susceptibility to hepatitis B in a representative sample of the pediatric and adolescent resident population in the province of Florence by analyzing the sera collected at the blood sampling center of the Meyer Children’s Hospital in Florence. Vaccination status or previous disease were also investigated. Another purpose of the study was to compare the obtained results with the data of the previous seroepidemiological surveys carried out in 2000 and 2009 within the same geographical area in order to describe the temporal trend of the immunity level against hepatitis B for the pediatric and adolescent population.

## 2. Materials and Methods

The collection of blood samples took place during the period December 2017–April 2018 at the blood sampling center of Meyer Children’s Hospital. All parents or guardians of the enrolled subjects gave a written consent for the inclusion before the enrollment. The study was conducted in accordance with the Declaration of Helsinki, and the protocol was approved by local ethics committees (Project identification code: DSS-UNIFI, n. registro pareri 98/2017).

Estimating the expected seroprevalence of anti-HBs equal to 60%, the calculated sample size was 164 sera. In a post-hoc analysis, we calculated the sample size to estimate the prevalence of the disease in the population with an accuracy of 7.5% and a confidence level of 95%. This sample size roughly represents 0.1% of the residents of Florence aged 1–18 years of a total population of 166,644 subjects in 2017 in the same age group [21], proportional to the population composition for each year-age group. Thus, no further standardization was required.

We excluded non-residents in the province of Florence, immunocompromised patients, subjects under immunosuppressive treatment, those with an acute infectious disease (measles, rubella, varicella, hepatitis A, and hepatitis B) in the previous two weeks, and those who had received a blood transfusion within the six months prior to the study [22,23,24,25,26,27,28,29,30].

All the collected blood samples were centrifuged (1600 rpm at 4 °C), and the recovered sera were stored at −20°C until tested for hepatitis B.

All the sera were tested for anti-HBs and total anti-HBc; any anti-HBs negative and total anti-HBc-positive serum sample would subsequently be tested for HBsAg.

The commercial ETI-AB-AUK-3 enzyme-linked immunosorbent Assay (ELISA) (Diasorin, Italy) was used to perform a quantitative measurement of anti-HBs; the antibody concentration ≥ 10 mIU/ml was considered indicative of a previous protective seroresponse. The commercial ETI-AB-COREK-PLUS enzyme-linked immunosorbent assay (ELISA) (Diasorin, Italy) was used to detect presence/absence of total anti-HBc in the samples. The criteria provided by the producer were applied for the serological evaluation of antibodies, according to the specific instruction manual. In case of equivocal sera, they would be tested twice in order to establish the positive or negative value, otherwise the sample would be considered equivocal. The positive samples for anti-HBs were divided into three different immunization levels: low (10–100 mIU/mL), intermediate (100–500 mIU/mL), and high (≥500 mIU/mL).

The National Registry of Notifications For Infectious Diseases (SIMI, software: Epi Info, Rome, Italy) was consulted to assess the anamnestic status of each subject. Through the vaccination registers SISPC (Collective Prevention Healthcare Information System) (Consortium Metis, Tuscany, Italy) and Caribel (Aster, Tuscany, Italy) (the current and the previously used vaccination coverage software in the Tuscany Region, respectively), we retrieved the vaccination status for hepatitis B, the number of anti-hepatitis B vaccine doses, the year of the last dose, and, when available, the type of the last administered vaccine for each enrolled subject.

The serological results were collected into an Excel database and assessed through a descriptive analysis for antibody titers, age group, and year of the last dose of vaccine received. To compare the ordinal variables (categorical groups of anti-HBs titers and age groups; categorical groups of anti-HBs titers and years-since-last-dose groups), the Spearman’s rank correlation coefficient was applied using STATA 12 (StataCorp, College Station, Texas, TX, USA). An ordinal logistic multivariate regression analysis of the enrolled population was performed using STATA 12, including the different categories of anti-HBs titers (negative: < 10 mIU/mL; low: 10–100 mIU/mL, intermediate: 100–500 mIU/mL and high: ≥ 500 mIU/mL) as a dependent variable, and “time since last dose”, “sex”, and “nationality” as the main independent variables. The variable “age” was not included, because it is co-linear with the “time since last dose” (correlation > 0.95). The *p*-value < 0.05 was considered statistically significant.

The serological profile towards hepatitis B obtained in the present study was compared with two previous serological surveys carried out in 2000 and 2009 within the area of Florence.

## 3. Results

The present study included 165 subjects aged 1–18 years for the serological analysis and for the evaluation of the anamnestic and vaccination status. For the serological descriptive analysis, the enrolled subjects were divided into four age groups as shown in Table 1. Males represented 53.3% of the study population, females—46.7%. Most of the enrolled subjects were Italian citizens (83%), the remaining part consisted of subjects with dual nationality (2.4%), subjects coming from Eastern Europe (Albania, Georgia, Romania, and Hungary) (5.5%), subjects coming from South America (Brazil and Peru) (1.8%), subjects coming from Africa (Cameroon, Morocco, Nigeria, and Senegal) (4.9%), and subjects coming from Asia (Bangladesh, Philippines, and Pakistan) (2.4%). The participants resided in 32 different districts of Florence, and about 48% of them were living in the City of Florence.

### 3.1. Confirmation of Vaccination and Anamnestic Status towards Hepatitis B

According to the vaccination registries, 155 of the enrolled subjects (94%) had received the anti-hepatitis B vaccine: 145 of them had completed the prime series of immunization (3 doses), 3 subjects had received a booster dose, and one child had received two booster doses. Therefore, six children did not complete the prime series of vaccination: one subject received only one dose of an anti-hepatitis B vaccine, and 5 subjects—two doses. Lastly, of the ten unvaccinated children, 4 were classified “protected” without any further specification.

Among the vaccinated subjects, 52 had received the hexavalent vaccine, and 3 children—the monovalent vaccine formulation. For the remaining subjects, no data on the specific vaccine product received was available.

Referring to the notification registry of infectious diseases, there were no reports of hepatitis B among the enrolled subjects.

### 3.2. Quantitave Analysis of Anti-HBs

**Figure 1:**
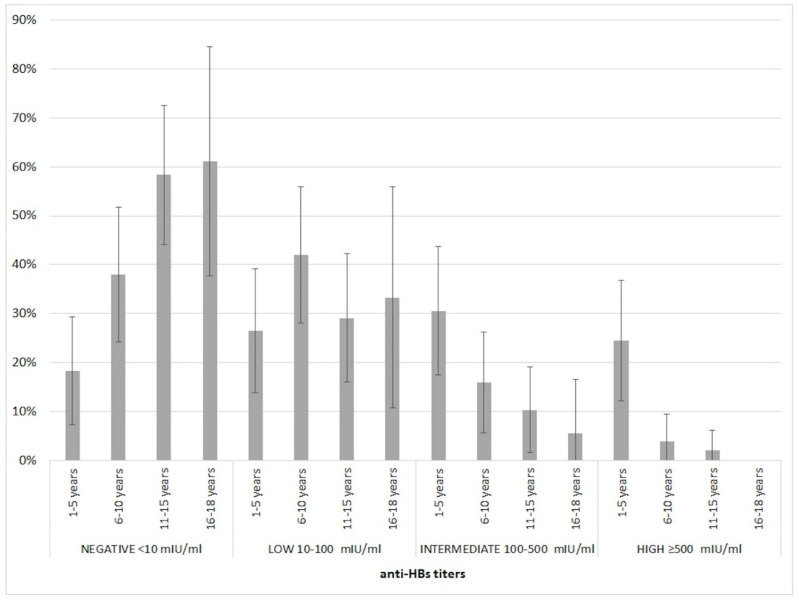
Percentage distribution of immunity levels in the total of subjects of different age groups.

About 59.4% of the tested samples (98/165) had anti-HBs ≥ 10mIU/mL. Taking age into account, adolescents of 16–18 years are characterized by a higher percentage (61.1%; 11/18) of subjects with no detectable anti-HBs. This value tends to decrease in the younger age groups, and reaches the minimum among children aged 1-5 years (18.4%; 9/49). Furthermore, none of the subjects aged 16–18 years had anti-HBs titers ≥ 500 mIU/mL. The Spearman’s rank correlation between the age group and the anti-HBs titer group is negative (−0.4316; *p* < 0.0001) (Table 2, Figure 1).

**Figure 2:**
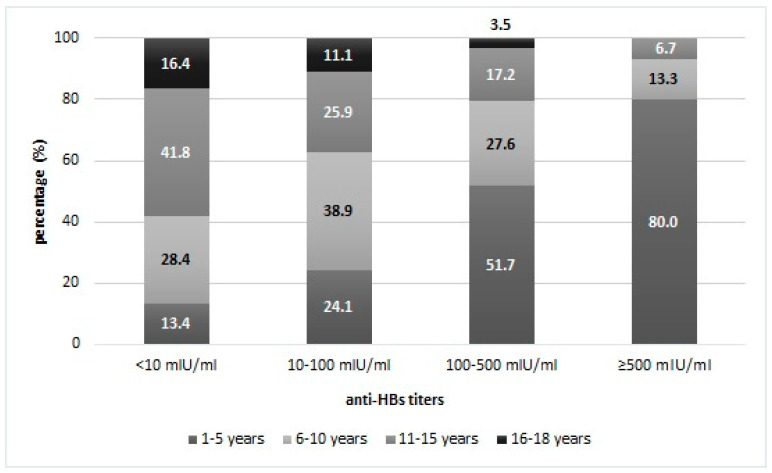
Percentage distribution of subjects of different age groups in the total of immunity levels.

Children 1–5-years had the highest anti-HBs titers, representing 80% of the samples with the highest anti-HBs concentration (≥500 mIU/mL). An opposite trend characterizes the older age groups; in particular, only 6.7% of adolescents aged 11–15 years had anti-HBs titers ≥ 500 mIU/mL, and all the adolescents aged 16–18 years had anti-HBs concentrations below 500 mIU/mL (Figure 2).

**Figure 3:**
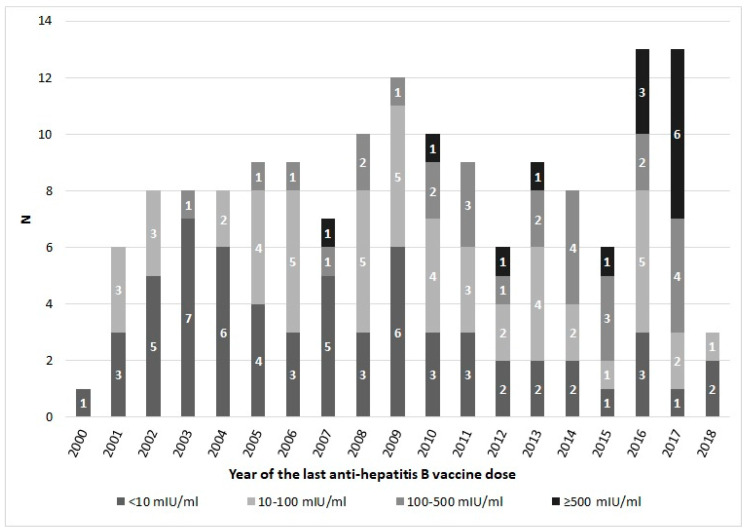
Immunity levels (anti-HBs) related to the years of the last anti-hepatitis B vaccine dose received.

Among the 149 subjects who received at least the prime immunization, 39% (58/149) had no detectable anti-HBs titers (<10 mIU/mL). Anti-HBs titers were detected in the subjects who had received the vaccination up to 16 years before (Figure 3). The Spearman’s correlation is negative (−0.3843; *p* < 0.0001) (Table 3).

### 3.3. Multivariate Analysis

The time elapsed since the last dose of a vaccine is significantly associated with the different degrees of anti-HBs titers adjusted for sex and nationality (Table 4). Sex and nationality have no significant effects on degrees of anti-HBs titers. Instead, the time since the last dose is predictive of the level of anti-HBs titers. For example, the children who had their immunization 6–10 years before had significantly lower antibody levels than the children who had received their last dose in the previous five years (adjusted odds ratio (AOR) = 0.35; *p* = 0.006).

### 3.4. Qualitative Analysis of Anti-HBc

All 165 collected sera were negative for anti-HBc. For this reason, we did not test for HBsAg in any sample.

### 3.5. Trend of Serological Profile towards Hepatitis B

The results of the present study can be compared with two similar seroepidemiological surveys carried out in 2000 [19] and 2009 [20] in the same area, respectively, 10 years and 20 years after implementation of the universal mandatory hepatitis B vaccination. In both studies, the samples were collected as residual sera from the blood sampling centers of two hospitals in Florence (the Meyer Children’s Hospital and the Careggi University Hospital) recording only the personal data related to sex and age. In both previous studies, the collected samples were representative not only of the city of Florence, but of the Tuscany Region in general, and the two study populations included individuals aged 1–50 years. Therefore, the comparison takes into account only the results obtained in the young population (1–10 and 11–20 year-olds).

As shown in Table 5, the seroprevalence of anti-HBs decreased from 2000 to 2009 in both age groups: in 1–10 year-olds, from 78.4% to 59.1%, and in 11–20 year-olds, from 82.4% to 58.1%, respectively. The current study (2018–2019) shows a similar proportion of subjects aged 1–10 years with anti-HBs titers *≥* 10 mIU/mL compared with the first study performed in 2000 (71.7% and 78.4%, respectively), but a lower percentage of anti-HBs-positive subjects among the adolescents aged 11–20 (40.9%) compared with the data of the two previous studies (in 2000: 82.4%; in 2009: 58.1%). Of great importance is the absence of anti-HBc among all the subjects of the present study compared with the results of the two previous studies.

## 4. Discussion

The study population is mainly represented by Italian subjects, only 17% of them has a dual nationality (Italian and foreign) or a foreign nationality. Most of the foreign subjects were born in Italy from foreign-born parents. Other subjects were adopted children born abroad. In this way, nationality does not necessarily imply a possible exposure to a high/intermediate endemic country for hepatitis B. Considering the general population, the estimated burden of migrants chronically infected with the hepatitis B virus from endemic countries accounts for 1–1.9 million residing in the European Union/European Economic Area (EU/EEA). Migrants from endemic countries represent 10.3% of the total EU/EEA population, contributing to 25% of all chronic hepatitis B cases. In particular, migrants born in China and Romania represent the largest number of estimated chronic infections [31]. In our study, no Chinese subjects were enrolled, and only three children had Romanian nationality: two of them were vaccinated against hepatitis B and had anti-HBs concentrations ≥ 10 mIU/mL. The other one, instead, was unvaccinated and negative for anti-HBs and anti-HBc. Furthermore, the multivariate analysis highlights that nationality is not associated with different levels of anti-HBs titers. Thus, considering all these factors, it is reasonable to consider foreign nationality a very low (almost zero) risk factor for hepatitis B in our study population.

Overall, seroprevalence of anti-HBs was approximately 60% in the study population. The children belonging to the first age group have the highest seropositivity rate (81.6%). The percentage of subjects with titers above the recommended threshold tends to decrease in the older age groups.

Among the 155 subjects who were vaccinated, 96% received the prime immunization course or booster doses: 39% of them had no detectable antibody titers, since after many years of vaccination the circulating antibodies tend to decrease below the protective threshold of 10 mIU/mL. As confirmed by the Spearman’s rank correlation and the multivariate analysis, the time elapsed since the last administration of an anti-hepatitis B vaccine can be associated with different degrees of immunity: the more recent the vaccination, the higher the concentration of circulating anti-HBs.

Although the positivity for anti-HBs tends to decrease over time, anti-HBs concentrations ≥ 10 mIU/ml were measured in the collected samples 16 years after the last anti-hepatitis B vaccine dose, and high antibody titers (≥500 mIU/mL)—up to 11 years after immunization.

As is it known, during the first year after the end of the prime immunization course, anti-HBs concentration tends to fall rather quickly, instead, in the following years, the decline is more gradual and slow [32,33].

Studies concerning the assessment of the persistence of protective antibody titers in the pediatric population have shown that 15–50% of responder subjects may have low or non-detectable antibody concentrations after 5–24 years since the vaccination [34,35,36]. The results obtained in the present study are, in this respect, in accordance with the collected data in the literature.

Although anti-HBs titers can decline below 10 mIU/mL after a complete prime vaccination series, this does not imply a loss of protection. In fact, the immune memory after anti-hepatitis B immunization continues to persist over a longer period [37,38]. The available data show that 67–76% of responder subjects who received prime childhood vaccine series (started at birth) are able to seroconvert after the administration of an additional booster dose up to 22 years after the prime immunization [39]. However, the absence of an anamnestic response following the booster dose may not necessarily mean that a subject is susceptible to the hepatitis B virus again [40]: the follow-up studies carried out to date have not shown disease (cases of acute or chronic infection) in this group of subjects [41,42]. The extensive literature regarding the immune memory developed following the vaccination may be extended to our study population.

The positivity for anti-HBs in five subjects who were unvaccinated according to the immunization registries is most probably due to a lack of registration of the vaccination, since none of them reports evidence of a previous infectious disease.

The absence of anti-HBc antibodies in the collected sera shows that the acquired immunity in the study population is mainly due to vaccination. This result, corroborated by the absence of reports of hepatitis B in the surveillance registries of infectious diseases for the enrolled subjects, seems to be in accordance with the decreasing trend of the reported cases in the last 10 years in Tuscany. Moreover, the obtained outcome is also in accordance with the low annual mean crude rates of reported cases that characterized children and young people aged 1–14 years (0.1–0.2/100,000 inhabitants) and adolescents and young adults aged 15–24 (0.6/100,000 inhabitants) in the period 2013–2017 [16].

Considering the serological profile trends described by the three different studies carried out in 2000, 2009, and 2019, the decrease in seroprevalence of anti-HBs between 2000 and 2009 could be due to the administration of the monovalent vaccine until 2003 instead of the hexavalent used since 2001 in the national immunization program [20]. Several studies have highlighted that one of the combined hexavalent vaccines used at first (subsequently withdrawn) was less immunogenic than the monovalent one and the other hexavalent vaccine. Nevertheless, DTaP-HBV-IPV-Hib vaccines induce lasting immune memory against the HBV, and the long-term protection is likely to be similar to that observed following priming with monovalent anti-HBV vaccines [43,44,45,46,47]. The percentage of anti-HBs positive subjects among the adolescents aged 11–20 years in the current study is lower compared with the data of the two previous studies. This could be explained considering the adolescents enrolled in 2017–2018 vaccinated during the first year of life, while the subjects aged ≥ 12 years (in the study of 2000) and the subjects aged 19–20 years had received the vaccination at 12 years (a double-cohort strategy during the period 1991–2003). Therefore, anti-HBs levels tend to be lower in the enrolled adolescents in the last serological survey (2017–2018) due to the greater time span from active immunization. Although this comparison may have some limits, like the different number of enrolled subjects for each age group and the different area of residence, the results highlight the important impact of the vaccination program 27 years after its implementation in Italy. In particular, the most relevant result concerns the immunity acquired by vaccination for all the enrolled subjects in the present study.

## 5. Conclusions

The introduction of the anti-HBV vaccination program in 1991 led to the change of the Italian epidemiological profile of hepatitis B, with a clear decrease of its prevalence and incidence, in particular among the young population, the target of the immunization programs.

The serological analysis showed that most enrolled subjects have anti-HBs titers ≥ 10 mIU/mL, and about 40% of vaccinated subjects are seronegative for anti-HBs. However, this percentage does not imply a susceptibility status for these subjects: the WHO affirms that even if the anti-HBs titers are not detectable following the immunization series, no booster dose is needed [1,2,3]. This is a consequence of the immune memory widely described for hepatitis B vaccination in the scientific literature [35,38].

The increasing trend of immunity levels consequent to active immunization among the pediatric and adolescent population confirms the importance of the anti-HBV vaccination 27 years after its implementation. Furthermore, the absence of anti-HBc in the collected sera highlights that all the enrolled subjects are immunized thanks to vaccination. This result is a further demonstration of how vaccination has helped to reduce the circulation of the hepatitis B virus in Tuscany in the pediatric and adolescent population.

Although the current study was carried out on a limited number of samples from a specific area of Italy, the obtained results can be the basis for considering the elimination of hepatitis B in Italy as an ever closer goal, since hepatitis B vaccination was introduced as a mandatory policy in all Italian regions, with homogeneous vaccination coverage in the last two decades.

## Figures and Tables

**Table 1:** Age groups of the study population.

Age Groups (years)	Enrolled Subjects (N)
1–5	49
6–10	50
11–15	48
16–18	18
TOTAL	165

**Table 2:** Summary of anti-HBs serological results in the study population.

Age (years)	Subjects (*N*; %)	<10 mIU/mL	10–100 mIU/mL	100–500 mIU/mL	≥500 mIU/mL	Total
1–5 years	*N*	9	13	15	12	49
	% [anti-HBs]	18.4	26.5	30.6	24.5	100
	% of the subjects	13.4	24.1	51.7	80.0	29.7
6–10 years	*N*	19	21	8	2	50
	% [anti-HBs]	38.0	42.00	16.0	4.0	100
	% of the subjects	28.4	38.9	27.6	13.3	30.3
11–15 years	*N*	28	14	5	1	48
	% [anti-HBs]	58.3	29.2	10.4	2.1	100
	% of the subjects	41.8	25.9	17.2	6.7	29.1
16–18 years	*N*	11	6	1	0	18
	% [anti-HBs]	61.1	33.3	5.6	0.0	100
	% of the subjects	16.4	11.1	3.5	0.0	10.9
	Total	67	54	29	15	165
		40.6	32.7	17.6	9.1	100
Spearman’s rho = −0.4316 *p* < 0.0001						

**Table 3:** Anti-HBs titer distribution related to the time elapsed since the last dose of an anti-hepatitis B vaccine received.

Time Since Last	Subjects (*N*; %)	<10	10–100	100–500	≥500	Total
dose (years)		mIU/mL	mIU/mL	mIU/mL	mIU/mL	
0–5	*N*	11	17	15	11	54
	% [anti-HBs]	20.4	31.5	27.8	20.4	100
	% of the subjects	16.4	31.5	51.7	73.3	32.7
6–10	*N*	18	18	9	2	47
	% [anti-HBs]	38.3	38.3	19.1	4.3	100
	% of the subjects	26.9	33.3	31.0	13.3	28.5
11–15	*N*	25	11	4	1	41
	% [anti-HBs]	61.0	26.8	9.8	2.4	100
	% of the subjects	37.3	20.4	13.8	6.7	24.8
16–18	*N*	8	5	0	0	13
	% [anti-HBs]	61.5	38.5	0.0	0.0	100
	% of the subjects	11.9	9.3	0.0	0.0	7.9
Unvaccinated	*N*	5	3	1	1	10
	% [anti-HBs]	50.0	30.0	10.0	10.0	100
	% of the subjects	7.5	5.6	3.4	6.7	6.1
	Total	67	54	29	15	165
		40.6	32.7	17.6	9.1	100
Spearman’s rho = −0.3843 *p* < 0.0001						

**Table 4:** Multivariate ordinal logistic regression analysis.

Dependent Variable: Degrees of Anti-HBs Titers					
		AOR	SE	95% CI	*p*-Value
**Sex**	Female	-	-	-	-
	Male	1.10	0.33	0.61-1.98	0.754
**Nationality**	Italian	-	-	-	-
	Foreign	1.44	0.65	0.59-3.51	0.415
**Time since the last dose**	0–5 years	-	-	-	-
	6–10 years	0.35	0.13	0.17–0.74	0.006
	11–15 years	0.15	0.06	0.06–0.35	< 0.0001
	16–18 years	0.13	0.08	0.04–0.43	0.001
	Unvaccinated	0.21	0.14	0.05–0.81	0.024

Note. AOR: Adjusted Odds Ratio. SE: Standard Error. CI: Confidential Interval. “-”: Reference.

**Table 5:** The summary of anti-HBs (≥10 mIU/mL), anti-HBc, and HBsAg prevalence for the three seroepidemiological studies carried out in Tuscany Region (2000, 2009) and in the province of Florence (2017–2018).

Studies(year)	Anti-HBs ≥ 10 mIU/mL (%)		Anti-HBc (%)		HBsAg (%)	
	1–10 years	11–20 years	1–10 years	11–20 years	1–10 years	11–20 years ^1^
2000	78.4	82.4	0	0.9	0	0
2009	59.1	58.1	4.1	5.0	0	0.6
2017–2018	71.7	40.9	0	0	0	0

^1^ Age group of 11–18 years for the study carried out in 2017–2018.
